# Gastric cardiac leiomyoma complicated with massive gastrointestinal bleeding: A rare case report

**DOI:** 10.1097/MD.0000000000043119

**Published:** 2025-07-04

**Authors:** Zhaohui Liu, Yaru Lei, Dayong Sun, Ruinuan Wu

**Affiliations:** a The Department of Gastroenterology, Shenzhen Second People’s Hospital, First Affiliated Hospital of Shenzhen University Health Science Center, Shenzhen, China; b Department of Medicine, Shenzhen University, Shenzhen, China; c The Department of Pathology, Shenzhen Second People’s Hospital, First Affiliated Hospital of Shenzhen University Health Science Center, Shenzhen, China.

**Keywords:** gastric cardia, gastrointestinal bleeding, leiomyoma, ultrasound endoscopy

## Abstract

**Rationale:**

We report a rare case of gastric cardiac leiomyoma with massive gastrointestinal bleeding as the first symptom that presented. This is the first case of this rare condition occurring in a patient younger than 20 years old. Positive results could not be obtained by routine biopsy, and the diagnosis was finally confirmed by endoscopic ultrasound-guided fine-needle aspiration biopsy.

**Patient concerns:**

A man aged less than 20 years old presented to the emergency department of the hospital due to upper gastrointestinal bleeding for 2 days.

**Diagnoses:**

Emergency gastroscopy revealed that a bulging mass could be seen at the gastric cardia. Computed tomography revealed a large hypoechoic mass in the cardia of the fundus without enhancement. Finally, endoscopic ultrasound-guided fine-needle aspiration biopsy was performed. The pathological diagnosis was a leiomyoma.

**Interventions:**

The patient ultimately opted for total gastrectomy.

**Outcomes:**

The patient’s gastric cardiac leiomyoma was completely resolved, and no digestive tract symptoms recurred after 1 month of follow-up.

**Lessons:**

For young patients with upper gastrointestinal bleeding, we should also be aware of the possibility of other rare causes. Endoscopic ultrasound-guided fine-needle aspiration is very valuable for the diagnosis of submucosal tumors.

## 1. Introduction

In young patients with acute upper gastrointestinal bleeding, the most common causes of bleeding are peptic ulcers and acute gastric mucosal lesions.^[1]^ Gastrointestinal bleeding caused by gastric muscularis propria tumors is more common in middle-aged and elderly patients, among which gastrointestinal stromal tumors are the most common tumor type and leiomyomas are rare.^[2]^ For young patients with upper gastrointestinal bleeding, doctors rarely associate leiomyomas with bleeding. This is the first case of a leiomyoma with massive hemorrhage in a young patient with a definitive diagnosis by endoscopic ultrasound-guided fine-needle aspiration.

## 2. Case report

**Figure 1.**
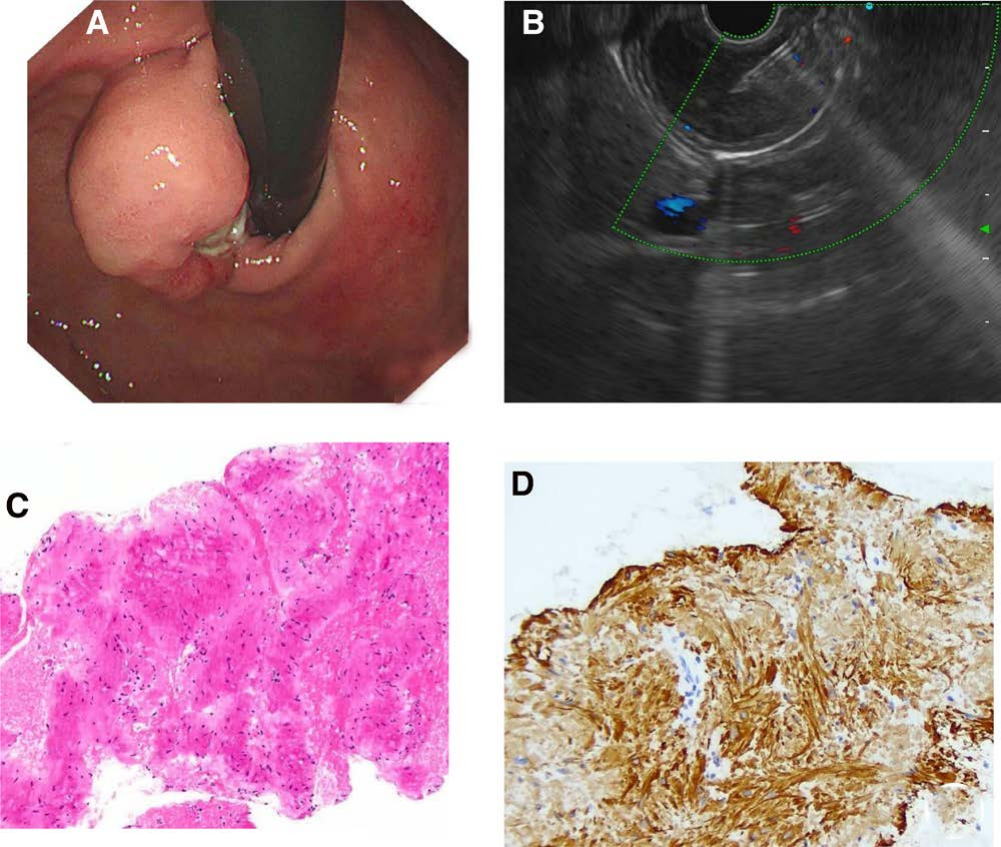
Endoscopic presentation. (A) The mass was found during white-light endoscopy. Ulcer formation was observed on the top of the mass. (B) Endoscopic ultrasound-guided fine-needle aspiration biopsy was performed. (C) The tumor cells are spindle-shaped with rod-shaped nuclei and deep staining. No mitotic figures were observed. (D) Immunohistochemical staining Desmin showed positive cytoplasm of tumor cells.

A male patient 19 years old who went to the emergency department of Shenzhen Second People’s Hospital for hematemesis and black stool. The patient experienced dizziness, fatigue and amaurosis. The results of routine blood tests revealed that the hemoglobin (HGB) level was 9.6 g/dL. The *Helicobacter pylori* was negative. We asked about the patient’s personal and family history. The patient had no family history of gastric tumors, and had no history of smoking or drinking. Proton pump inhibitors and somatostatin were used for initial drug therapy. We performed emergency gastroscopy on the patient. Gastroscopy revealed that a bulging mass could be seen at the gastric cardia. Ulcer formation was observed on the top of the mass, and other parts of the mass were covered with normal mucosa with no active bleeding (Fig. 1A). The operator retrieved the biopsy sample from beside the ulcer, and the pathology suggested chronic mucosal inflammation. The patient underwent enhanced CT, which revealed a large low density mass in the cardia of the fundus without enhancement. The EUS indicated that the tumor originated from the muscularis propria, with hypo-echoic and uniform internal echo. Finally, endoscopic ultrasound-guided fine-needle aspiration biopsy was performed (Fig. 1B). The pathological diagnosis was a leiomyoma (Fig. 1C and D). The patient ultimately opted for total gastrectomy.

The reporting of this study conforms to CARE guidelines. In addition, we obtained board approval for publication. The study was conducted in accordance with the 2008 revision of the Helsinki Declaration and was approved by the Ethics Committee of the Second People’s Hospital of Shenzhen Municipality.

## 3. Discussion

The most common causes of upper gastrointestinal bleeding are peptic ulcers, esophageal and gastric fundus varices, gastric cancer, acute bleeding and erosive gastritis.^[3–6]^ In young patients, the most common disease causing gastrointestinal bleeding is peptic ulcers.^[7]^ When the amount of bleeding exceeds 800 mL, the patient will experience clinical symptoms of circulatory disorders, such as dizziness, fatigue, and syncope.^[8]^ In this case, the patient developed the above symptoms while bleeding, indicating serious bleeding, as confirmed by blood test results.

The guidelines recommend that patients with acute upper gastrointestinal bleeding need to complete emergency gastroscopy within 24 hours of admission so that the cause of the bleeding can be identified as soon as possible and endoscopic hemostasis can be performed if necessary.^[9]^ In this patient, emergency gastroscopy was performed within 24 hours after admission. On the basis of previous diagnosis and treatment experience and evidence-based medical evidence, we considered that the patient was more likely to have peptic ulcers, but the gastroscopy results were unexpected.

Gastrointestinal bleeding caused by a gastric leiomyoma is extremely rare. At present, the reported cases all occurred in middle-aged or elderly people.^[10,11]^ This is the first case report of a gastric leiomyoma occurring in a patient younger than 20 years of age. The patient did not have any gastrointestinal symptoms before onset, and the first symptom was massive upper gastrointestinal bleeding. This finding also suggests the necessity of gastroscopy. The ulcer may be caused by the rapid growth of the tumor, which leads to ischemic necrosis of the mucosa attached to the surface of the tumor. The etiology of gastric leiomyoma is not yet fully understood. It is currently believed that it may be related to a variety of factors, such as family genetic predisposition, gene mutations, elevated estrogen levels, and environmental factors.

For gastric submucosal tumors, endoscopic biopsy often fails to obtain a satisfactory pathological diagnosis because most of these tumors originate from the muscularis propria, and biopsy can only be performed on the submucosa but not the muscularis propria. Endoscopic ultrasound-guided fine-needle aspiration solves this problem well.^[12]^ The patient’s final diagnosis was confirmed via ultrasound-guided fine-needle biopsy. Although the leiomyoma is a benign tumor, a leiomyoma with concurrent bleeding has surgical indications; thus, the patient ultimately chose surgical treatment, and no digestive tract symptoms recurred after 1 month of follow-up.

This is a rare case of gastrointestinal bleeding that occurred in a young patient with gastric cardia leiomyoma. We clarified the diagnosis by endoscopic ultrasound-guided fine-needle aspiration biopsy. Therefore, for young patients with upper gastrointestinal bleeding, we should also be aware of the possibility of other rare causes.

There is a limitation in this work. Since this is a rare case report, it cannot represent the universality of clinical practice, so it should be combined with specific circumstances in clinical practice.

## Acknowledgments

The authors thank the anonymous reviewers and the editor for their valuable comments.

## Author contributions

**Data curation:** Yaru Lei.

**Funding acquisition:** Dayong Sun.

**Investigation:** Zhaohui Liu, Ruinuan Wu.

**Resources:** Zhaohui Liu, Yaru Lei.

**Supervision:** Dayong Sun, Ruinuan Wu.

**Writing – original draft:** Zhaohui Liu.

**Writing – review & editing:** Ruinuan Wu.
